# Relationship between the Clinical Frailty Scale and short-term mortality in patients ≥ 80 years old acutely admitted to the ICU: a prospective cohort study

**DOI:** 10.1186/s13054-021-03632-3

**Published:** 2021-07-01

**Authors:** Jakub Fronczek, Kamil Polok, Dylan W. de Lange, Christian Jung, Michael Beil, Andrew Rhodes, Jesper Fjølner, Jacek Górka, Finn H. Andersen, Antonio Artigas, Maurizio Cecconi, Steffen Christensen, Michael Joannidis, Susannah Leaver, Brian Marsh, Alessandro Morandi, Rui Moreno, Sandra Oeyen, Christina Agvald-Öhman, Bernardo Bollen Pinto, Joerg C. Schefold, Andreas Valentin, Sten Walther, Ximena Watson, Tilemachos Zafeiridis, Sigal Sviri, Peter Vernon van Heerden, Hans Flaatten, Bertrand Guidet, Wojciech Szczeklik, for the VIP1, R. Schmutz, F. Wimmer, P. Eller, M. Joannidis, P. De Buysscher, N. De Neve, S. Oeyen, W. Swinnen, B. Bollen Pinto, P. Abraham, L. Hergafi, J. C. Schefold, E. Biskup, P. Piza, I. Taliadoros, J. Fjølner, N. Dey, C. Sølling, B. S. Rasmussen, S. Christensen, X. Forceville, G. Besch, H. Mentec, P. Michel, P. Mateu, P. Michel, L. Vettoretti, J. Bourenne, N. Marin, M. Guillot, N. Aissaoui, C. Goulenok, N. Thieulot-Rolin, J. Messika, L. Lamhaut, B. Guidet, C. Charron, A. Lauten, A. L. Sacher, T. Brenner, M. Franz, F. Bloos, H. Ebelt, S. J. Schaller, K. Fuest, C. Rabe, T. Dieck, S. Steiner, T. Graf, A. M. Nia, C. Jung, R. A. Janosi, P. Meybohm, P. Simon, S. Utzolino, T. Rahmel, E. Barth, C. Jung, M. Schuster, Z. Aidoni, S. Aloizos, P. Tasioudis, K. Lampiri, V. Zisopoulou, I. Ravani, E. Pagaki, A. Antoniou, T. A. Katsoulas, A. Kounougeri, G. Marinakis, F. Tsimpoukas, A. Spyropoulou, P. Zygoulis, A. Kyparissi, M. Gupta, M. Gurjar, I. M. Maji, I. Hayes, B. Marsh, Y. Kelly, A. Westbrook, G. Fitzpatrick, D. Maheshwari, C. Motherway, G. Negri, S. Spadaro, G. Nattino, M. Pedeferri, A. Boscolo, S. Rossi, G. Calicchio, L. Cubattoli, G. Di Lascio, M. Barbagallo, F. Berruto, D. Codazzi, A. Bottazzi, P. Fumagalli, G. Negro, G. Lupi, F. Savelli, G. A. Vulcano, R. Fumagalli, A. Marudi, U. Lefons, R. Lembo, M. Babini, A. Paggioro, V. Parrini, M. Zaccaria, S. Clementi, C. Gigliuto, F. Facondini, S. Pastorini, S. Munaron, I. Calamai, A. Bocchi, A. Adorni, M. G. Bocci, A. Cortegiani, T. Casalicchio, S. Mellea, E. Graziani, M. Barattini, E. Brizio, M. Rossi, M. Hahn, H. Flaatten, N. Kemmerer, H. F. Strietzel, K. Dybwik, T. Legernaes, P. Klepstad, E. B. Olaussen, K. I. Olsen, O. M. Brresen, G. Bjorsvik, F. H. Andersen, S. Maini, L. Fehrle, M. Czuczwar, P. Krawczyk, M. Ziętkiewicz, Ł. R. Nowak, K. Kotfis, K. Cwyl, R. Gajdosz, J. Biernawska, R. Bohatyrewicz, R. Gawda, P. Grudzień, P. Nasiłowski, N. Popek, W. Cyrankiewicz, K. Wawrzyniak, M. Wnuk, D. Maciejewski, D. Studzińska, M. Żukowski, S. Bernas, M. Piechota, W. Szczeklik, I. Nowak-Kózka, J. Fronczek, M. Serwa, W. Machała, J. Stefaniak, M. Wujtewicz, P. Maciejewski, M. Szymkowiak, B. Adamik, K. Polok, J. Górka, N. Catorze, M. C. Branco, N. Barros, I. Barros, A. Krystopchuk, T. Honrado, C. Sousa, F. Munoz, M. Rebelo, R. Gomes, J. Nunes, C. Dias, A. M. Fernandes, C. Petrisor, B. Constantin, V. Belskiy, B. Boskholov, E. Rodriguez, G. Aguilar, G. Masdeu, M. I. Jaimes, A. P. Mira, M. A. Bodi, J. A. B. Mendoza, S. López-Cuenca, M. H. Guzman, J. Rico-Feijoo, M. Ibarz, J. Trenado Alvarez, R. Kawati, J. Sivik, J. Nauska, D. Smole, F. Parenmark, J. Lyrén, K. Rockstroh, S. Rydén, M. Spångfors, M. Strinnholm, S. Walther, L. De Geer, P. Nordlund, S. Pålsson, H. Zetterquist, A. Nilsson, K. Thiringer, M. Jungner, B. Bark, B. Nordling, H. Sköld, C. Brorsson, S. Persson, A. Bergström, J. Berkius, J. Holmström, I. van Dijk, L. E. M. van Lelyveld-Haas, T. Jansen, F. Nooteboom, P. H. J. van der Voort, D. de Lange, W. Dieperink, M. C. de Waard, A. G. E. de Smet, L. Bormans, T. Dormans, G. Dempsey, S. J. Mathew, A. S. Raj, I. Grecu, J. Cupitt, T. Lawton, R. Clark, M. Popescu, N. Spittle, M. Faulkner, A. Cowton, P. Williams, E. Elloway, M. Reay, S. Chukkambotla, R. Kumar, N. Al-Subaie, L. Kent, T. Tamm, I. Kajtor, K. Burns, R. Pugh, M. Ostermann, E. Kam, H. Bowyer, N. Smith, M. Templeton, J. Henning, K. Goffin, R. Kapoor, S. Laha, P. Chilton, W. Khaliq, A. Crayford, S. Coetzee, M. Tait, W. Stoker, M. Gimenez, A. Pope, J. Camsooksai, D. Pogson, K. Quigley, J. Ritzema, A. Hormis, C. Boulanger, M. Balasubramaniam, L. Vamplew, K. Burt, D. Martin, I. Grecu, J. Craig, J. Prowle, N. Doyle, J. Shelton, C. Scott, P. Donnison, S. Shelton, C. Frey, C. Ryan, D. Spray, C. Ryan, V. Barnes, K. Barnes, S. Ridgway, R. Saha, L. Kent, T. Clark, J. Wood, C. Bolger, C. Bassford, A. Cowton, J. Lewandowski, X. Zhao, S. Humphreys, S. Dowling, N. Richardson, A. Burtenshaw, C. Stevenson, D. Wilcock, Y. Nalapko, VIP2 study group, M. Joannidis, P. Eller, R. Helbok, R. Schmutz, J. Nollet, N. de Neve, P. De Buysscher, S. Oeyen, W. Swinnen, M. Mikačić, A. Bastiansen, A. Husted, B. E. S. Dahle, C. Cramer, C. Sølling, D. Ørsnes, J. Edelberg Thomsen, J. J. Pedersen, M. Hummelmose Enevoldsen, T. Elkmann, A. Kubisz-Pudelko, A. Pope, A. Collins, A. S. Raj, C. Boulanger, C. Frey, C. Hart, C. Bolger, D. Spray, G. Randell, H. Filipe, I. D. Welters, I. Grecu, J. Evans, J. Cupitt, J. Lord, J. Henning, J. Jones, J. Ball, J. North, K. Salaunkey, L. Ortiz-Ruiz De Gordoa, L. Bell, M. Balasubramaniam, M. Vizcaychipi, M. Faulkner, M. Mupudzi, M. Lea-Hagerty, M. Reay, M. Spivey, N. Love, N. Spittle, N. White, P. Williams, P. Morgan, P. Wakefield, R. Savine, R. Jacob, R. Innes, R. Kapoor, S. Humphreys, S. Rose, S. Dowling, S. Leaver, T. Mane, T. Lawton, V. Ogbeide, W. Khaliq, Y. Baird, A. Romen, A. Galbois, B. Guidet, C. Vinsonneau, C. Charron, D. Thevenin, E. Guerot, G. Besch, G. Savary, H. Mentec, J. L. Chagnon, J. P. Rigaud, J. P. Quenot, J. Castaneray, J. Rosman, J. Maizel, K. Tiercelet, L. Vettoretti, M. M. Hovaere, M. Messika, M. Djibré, N. Rolin, P. Burtin, P. Garcon, S. Nseir, X. Valette, C. Rabe, E. Barth, H. Ebelt, K. Fuest, M. Franz, M. Horacek, M. Schuster, P. Meybohm, R. Romano Bruno, S. Allgäuer, S. Dubler, S. J. Schaller, S. Schering, S. Steiner, T. Dieck, T. Rahmel, T. Graf, A. Koutsikou, A. Vakalos, B. Raitsiou, E. N. Flioni, E. Neou, F. Tsimpoukas, G. Papathanakos, G. Marinakis, I. Koutsodimitropoulos, K. Aikaterini, N. Rovina, S. Kourelea, T. Polychronis, V. Zidianakis, V. Konstantinia, Z. Aidoni, B. Marsh, C. Motherway, C. Read, I. Martin-Loeches, A. Neville Cracchiolo, A. Morigi, I. Calamai, S. Brusa, A. Elhadi, A. Tarek, A. Khaled, H. Ahmed, W. Ali Belkhair, A. D. Cornet, D. Gommers, D. de Lange, E. van Boven, J. Haringman, L. Haas, L. van den Berg, O. Hoiting, P. de Jager, R. T. Gerritsen, T. Dormans, W. Dieperink, A. Breidablik, A. Slapgard, A. K. Rime, B. Jannestad, B. Sjøbøe, E. Rice, F. H. Andersen, H. F. Strietzel, J. P. Jensen, J. Langørgen, K. Tøien, K. Strand, M. Hahn, P. Klepstad, A. Biernacka, A. Kluzik, B. Kudlinski, D. Maciejewski, D. Studzińska, H. Hymczak, J. Stefaniak, J. Solek-Pastuszka, J. Zorska, K. Cwyl, Ł. J. Krzych, M. Zukowski, M. Lipińska-Gediga, M. Pietruszko, M. Piechota, M. Serwa, M. Czuczwar, M. Ziętkiewicz, N. Kozera, P. Nasiłowski, P. Sendur, P. Zatorski, P. Galkin, R. Gawda, U. Kościuczuk, W. Cyrankiewicz, W. Gola, A. F. Pinto, A. M. Fernandes, A. R. Santos, C. Sousa, I. Barros, I. A. Ferreira, J. B. Blanco, J. T. Carvalho, J. Maia, N. Candeias, N. Catorze, V. Belskiy, A. Lores, A. P. Mira, C. Cilloniz, D. Perez-Torres, E. Maseda, E. Rodriguez, E. Prol-Silva, G. Eixarch, G. Gomà, G. Aguilar, G. Navarro Velasco, M. Irazábal Jaimes, M. Ibarz Villamayor, N. Llamas Fernández, P. Jimeno Cubero, S. López-Cuenca, T. Tomasa, A. Sjöqvist, C. Brorsson, F. Schiöler, H. Westberg, J. Nauska, J. Sivik, J. Berkius, K. Kleiven Thiringer, L. De Geer, S. Walther, F. Boroli, J. C. Schefold, L. Hergafi, P. Eckert, I. Yıldız, I. Yovenko, Y. Nalapko, R. Pugh

**Affiliations:** 1 Department of Medicine, Center for Intensive Care and Perioperative Medicine, Jagiellonian University Medical College, ul. Skawińska 8, 31 – 066 Kraków, Poland; 2 Department of Intensive Care Medicine, University Medical Center, University Utrecht, Utrecht, The Netherlands; 3 Division of Cardiology, Pulmonology and Vascular Medicine, University Hospital Düsseldorf, Heinrich-Heine-University, Düsseldorf, Germany; 4 Medical Intensive Care Unit, Hadassah Medical Center, Jerusalem, Israel; 5 St George’s University Hospitals NHS Foundation Trust, London, London, UK; 6 Department of Intensive Care, Aarhus University Hospital, Århus, Denmark; 7 Department of Anaesthesia and Intensive Care, Ålesund Hospital, Ålesund, Norway; 8 Department of Circulation and Medical Imaging, NTNU, Trondheim, Norway; 9 Critical Care Department, Corporacion Sanitaria Universitaria Parc Tauli, CIBER Enfermedades Respiratorias, Autonomous University of Barcelona, Sabadell, Spain; 10 Department of Anesthesia and Intensive Care Medicine, Humanitas Clinical and Research Center – IRCCS, Via Alessandro Manzoni 56, 20089 Rozzano, MI Italy; 11 Department of Biomedical Sciences, Humanitas University, Pieve Emanuele, Rozzano, MI Italy; 12 Division of Intensive Care and Emergency Medicine, Department of Internal Medicine, Medical University Innsbruck, Innsbruck, Austria; 13 Research Lead Critical Care Directorate St George’s Hospital, London, UK; 14 Mater Misericordiae University Hospital, Dublin, Ireland; 15 Department of Rehabilitation Hospital Ancelle di Cremona Italy, Geriatric Research Group, Brescia, Italy; 16 Faculdade de Ciências Médicas de Lisboa (Nova Médical School), Unidade de Cuidados Intensivos Neurocríticos e Trauma, Hospital de São José, Centro Hospitalar Universitário de Lisboa Central, Lisbon, Portugal; 17 Department of Intensive Care 1K12IC, Ghent University Hospital, Ghent, Belgium; 18 Karolinska University Hospital, Stockholm, Sweden; 19 Department of Anaesthesiology, Pharmacology and Intensive Care, Geneva University Hospitals, Geneva, Switzerland; 20 Department of Intensive Care Medicine, Inselspital, Bern University Hospital, University of Bern, Bern, Switzerland; 21 Kardinal Schwarzenberg Hospital, Schwarzach, Austria; 22 Department of Cardiothoracic Surgery, Anesthesia and Intensive Care, Linköping University Hospital and Department of Medical and Health Sciences, Linköping University, Linköping, Sweden; 23 Intensive Care Unit, General Hospital of Larissa, Larissa, Greece; 24 Department of Medical Intensive Care, Hadassah Medical Center and Faculty of Medicine, Hebrew University of Jerusalem, Jerusalem, Israel; 25 Department of Anesthesia, Intensive Care and Pain Medicine, Hadassah Medical Center and Faculty of Medicine, Hebrew University of Jerusalem, Jerusalem, Israel; 26 Department of Anaesthesia and Intensive Care, Haukeland University Hospital, Bergen, Norway; 27 Department of Clinical Medicine, University of Bergen, Bergen, Norway; 28 UPMC Univ Paris 06, INSERM, UMR_S 1136, Institut Pierre Louis d’Epidémiologie et de Santé Publique, Equipe: Epidémiologie Hospitalière Qualité et Organisation des Soins, Sorbonne Universités, Assistance Publique - Hôpitaux de Paris, 75012 Paris, France

**Keywords:** Intensive care units, Aged, 80 and over, Frailty, Prospective studies, Mortality

## Abstract

**Background:**

The Clinical Frailty Scale (CFS) is frequently used to measure frailty in critically ill adults. There is wide variation in the approach to analysing the relationship between the CFS score and mortality after admission to the ICU. This study aimed to evaluate the influence of modelling approach on the association between the CFS score and short-term mortality and quantify the prognostic value of frailty in this context.

**Methods:**

We analysed data from two multicentre prospective cohort studies which enrolled intensive care unit patients ≥ 80 years old in 26 countries. The primary outcome was mortality within 30-days from admission to the ICU. Logistic regression models for both ICU and 30-day mortality included the CFS score as either a categorical, continuous or dichotomous variable and were adjusted for patient’s age, sex, reason for admission to the ICU, and admission Sequential Organ Failure Assessment score.

**Results:**

The median age in the sample of 7487 consecutive patients was 84 years (IQR 81–87). The highest fraction of new prognostic information from frailty in the context of 30-day mortality was observed when the CFS score was treated as either a categorical variable using all original levels of frailty or a nonlinear continuous variable and was equal to 9% using these modelling approaches (*p* < 0.001). The relationship between the CFS score and mortality was nonlinear (*p* < 0.01).

**Conclusion:**

Knowledge about a patient’s frailty status adds a substantial amount of new prognostic information at the moment of admission to the ICU. Arbitrary simplification of the CFS score into fewer groups than originally intended leads to a loss of information and should be avoided.

*Trial registration* NCT03134807 (VIP1), NCT03370692 (VIP2)

**Supplementary Information:**

The online version contains supplementary material available at 10.1186/s13054-021-03632-3.

## Introduction

Progressive ageing is one of the leading issues in contemporary critical care [1]. Patients ≥ 80 years old account for 10 to 20% of all admissions to the intensive care unit (ICU) , and this number is projected to increase sharply over next 3 decades [2]. The growing proportion of older adults admitted to ICUs will require substantial resources, at the same time as many countries are already facing a shortage of ICU beds [3–5]. The high mortality in the population of older critically ill adults has prompted researchers to identify factors which could help improve the allocation of scarce intensive care resources and alleviate the suffering of patients and their families facing an uncertain prognosis [6].

Among many geriatric syndromes, frailty may be the most useful concept in the context of severe illness requiring admission to the ICU [7]. Frailty is defined as a clinically recognisable state of increased vulnerability resulting from ageing-associated decline in reserve and function across multiple physiologic systems such that the ability to cope with acute stressors is compromised [8, 9]. The presence of frailty is associated with increased morbidity in older adults, and the chances of 30-day survival after admission to the ICU are significantly lower in frail patients compared to their fit counterparts [10–12].

The Clinical Frailty Scale (CFS) is a widely used frailty assessment tool which has gained recognition in a variety of clinical settings over the past decade [13]. Unlike many other instruments describing the degree of frailty, the CFS score was shown to be reliable in the highly dynamic context of acute admission to the ICU in patients ≥ 80 years old [14, 15]. Frailty is most often defined as a CFS score greater than or equal to 5, with a CFS score of 4 labelled as pre-frailty (vulnerability, living with mild frailty) and CFS scores between 1 and 3 referred to as fitness. However, recent evidence shows that no clinically relevant worsening in prognosis occurs at the transition of pre-frailty to frailty, suggesting that such categorisation does not accurately reflect the relationship between the CFS and patients’ outcomes [16]. Analysing the CFS score as a continuous variable provides a solution to the problem of arbitrary stratification of patients according to pre-defined thresholds, but at the same time makes a strong assumption that the risk of an outcome of interest increases linearly with increasing levels of frailty.

The objective of this study was to establish the optimal approach to analysing the association between pre-admission frailty and short-term mortality by means of the CFS score using a large sample of very old intensive care unit patients (VIPs) acutely admitted to the ICU within two international prospective cohort studies. We aimed to formally test the hypothesis that the association between frailty and ICU mortality is linear, describe the risk of ICU mortality across the entire spectrum of CFS scores, and quantify the added prognostic value of frailty in patients ≥ 80 years old.

## Methods

### Study design and patient population

The Very Old Intensive Care Patient (VIP1 and VIP2) studies were prospective, multicentre studies registered on ClinicalTrials.gov (ID: NCT03134807, NCT03370692, respectively) [7, 11]. Both studies included patients aged 80 years or older admitted to the ICU with no specific exclusion criteria, and all acutely admitted patients were eligible for this analysis. Sources of data, methods of measurement, and results of analyses performed previously on these data were described in detail elsewhere [7, 11]. In summary, all participating ICUs were asked to include 20 consecutive VIPs over a 3-month (VIP1) or 6-month (VIP2) period. Patients’ vital status within 30 days of admission to the ICU was assessed by inspecting hospital records, direct contact with the patient, or checking a national registry. Patients were recruited between October 2016 and February 2017 (VIP1 study) and between May 2018 and May 2019 (VIP2 study). All procedures performed in studies involving human participants were in accordance with the ethical standards of the institutional and/or national research committee and with the 1964 Declaration of Helsinki and its later amendments or comparable ethical standards. Each country had a national coordinator responsible for application for national or regional ethical and regulatory study approval. Institutional research ethic board approval was obtained from each study site. Some countries were allowed to recruit patients without prior written informed consent, while others had to collect informed consent from patient or their legal representative.

### Outcomes and procedure

Outcomes in this study were ICU mortality and 30-day mortality. Demographic data which included patients’ age, sex, and reason for admission to the ICU were collected in each eligible participant. Severity of organ dysfunction within the first 24 h after admission to the ICU was assessed using the cumulative Sequential Organ Failure Assessment (SOFA) score (from 0 to 24 points assigned in the cardiovascular, respiratory, renal, neurologic, hepatic, and coagulation system with increasing number of points corresponding to more severe organ failure; the highest summary score observed within the first 24 h was reported) [17]. Necessity for organ support (vasoactive drugs, intubation and mechanical ventilation, and renal replacement therapy) was documented during the ICU stay.

We used the Clinical Frailty Scale (CFS) to describe patients’ frailty before admission to the hospital with 9 possible classes from very fit prior to the acute illness to terminally ill [18]. Information necessary to perform this assessment was given by patients or proxy or obtained from patient records. The CFS visual along with a simple description was used with permission. We performed separate analyses using the 8-point variant of the scale (from fit to very severely frail) and the 9-point scale, where patients in the last category are not expected to survive more than 6 months but are not otherwise evidently frail. Primary analyses were based on the 8-point version of this tool [16].

### Statistical analysis

We used the likelihood ratio test to answer the question whether frailty adds new information about the risk of death in the ICU and at 30-day follow-up to baseline variables available at admission in a logistic regression model. (i.e. age, sex, reasons for admission to the ICU, and the SOFA score) [19]. Statistical models were built based on clinical rationale and utilised all baseline variables available in the merged dataset. We decided to perform a complete-case analysis as the overall amount of missing data was relatively small. A detailed inspection of missing data was performed and presented in Additional file 1.

We used five different approaches to analysing frailty in logistic regression models:

1. *Categorical:* we modelled the CFS score as a categorical variable using all original levels (i.e. 8 or 9 categories in the main and supplementary analysis, respectively).
2. *Linear:* CFS was treated as a continuous variable without allowing for nonlinearities in the log-odds for mortality (i.e. a standard technique used as a default setting in statistical software).
3. *Nonlinear:* the relationship between the CFS and the outcome was modelled using restricted cubic splines with five knots at the 5th, 27.5th, 50th, 72.5th, and 95th quantile of the distribution of CFS, thus allowing for nonlinear relationships and avoiding the known problems resulting from categorization of continuous variables (e.g. loss of statistical power, increased risk of false positive findings, underestimation of variation in outcomes between groups) [20]. Wald tests were used to assess departures from linearity of log-odds.
4. *Simplified grouping:* we labelled patients as fit (CFS < 4), pre-frail (CFS = 4), or frail (CFS ≥ 5) and constructed the model using such created categorical variable with three levels.
5. *Dichotomisation:* we defined frailty using a single threshold (CFS ≥ 5) and included a binary indicator of patient’s frailty in the model.

The reference category for calculating odds ratios (ORs) was CFS = 1 for the categorical, linear, and nonlinear approach. Simplified grouping and dichotomisation used CFS < 4 and CFS < 5 as a reference to calculate ORs, respectively.

Age was modelled using restricted cubic splines with four knots at the 5th, 35th, 65th, and 95th quantile of its distribution in all analyses. We summarized the added prognostic value of frailty as the fraction of new information (proportion of explainable variation that is explained by frailty or ratio of variances of predicted values before and after adding frailty to the model containing only baseline variables described above) [19]. Descriptive statistics on baseline variables are presented as median (interquartile range [IQR]) or count and percentage. All analyses were performed using R version 3.6.0 (R Project).

## Results

The combined number of acutely admitted patients in both the VIP 1 and VIP 2 studies was equal to 8173. The median number of subjects recruited per country was 166 (IQR 33–482), and the two most actively recruiting countries were UK (1619) and France (971). Survival status within 30 days from admission to the ICU was known in 94.5% of the patients, and 99.5% of the patients had complete data on ICU survival. Among patients whose survival status was known, 96.9% had complete data on all remaining variables. Patterns of missing data as well as a comparison of patients with complete and incomplete data on survival status are provided in Additional file 1: Figures S1–S3 and Tables S1 and S2.

Descriptive statistics, overall and among different categories of the CFS score of the analysed sample (*n* = 7487), are presented in Table 1. Patients classified as very frail (CFS = 8) and terminally ill (CFS = 9) had the highest SOFA scores and the highest incidence of vasoactive drugs and mechanical ventilation initiation compared to participants with lower CFS scores. The length of stay was by far the longest in very frail patients who survived until discharge from the ICU.

**Table 1:** Sample characteristics

Variable	Overall	CFS = 1	CFS = 2	CFS = 3	CFS = 4	CFS = 5	CFS = 6	CFS = 7	CFS = 8	CFS = 9
*n*	7487	319	756	1723	1446	1097	1127	730	246	43
Male sex (*n*, %)	3934 (52.5)	200 (62.7)	455 (60.2)	988 (57.3)	783 (54.1)	530 (48.3)	504 (44.7)	333 (45.6)	122 (49.6)	19 (44.2)
Age, years (median [IQR])	84.0 [81.0, 87.0]	82.0 [81.0, 85.0]	83.0 [81.0, 86.0]	83.0 [81.0, 86.0]	84.0 [81.0, 86.0]	84.0 [82.0, 87.0]	84.0 [82.0, 88.0]	85.0 [82.0, 88.0]	84.0 [82.0, 87.0]	83.0 [81.0, 87.0]
SOFA score (median [IQR])	7.0 [4.0, 10.0]	6.0 [3.0, 8.0]	6.0 [4.0, 10.0]	6.0 [4.0, 10.0]	6.0 [4.0, 10.0]	7.0 [4.0, 10.0]	7.0 [4.0, 10.0]	8.0 [5.0, 10.0]	9.0 [6.0, 12.0]	9.0 [7.5, 12.5]
Reason for admission (*n*, %)										
Respiratory failure	1809 (24.2)	67 (21.0)	154 (20.4)	358 (20.8)	374 (25.9)	278 (25.3)	300 (26.6)	191 (26.2)	73 (29.7)	14 (32.6)
Circulatory failure	1057 (14.1)	32 (10.0)	111 (14.7)	240 (13.9)	221 (15.3)	157 (14.3)	166 (14.7)	100 (13.7)	23 (9.3)	7 (16.3)
Combined respiratory/circulatory failure	874 (11.7)	17 (5.3)	58 (7.7)	165 (9.6)	183 (12.7)	137 (12.5)	141 (12.5)	116 (15.9)	50 (20.3)	7 (16.3)
Sepsis	992 (13.2)	26 (8.2)	80 (10.6)	242 (14.0)	159 (11.0)	162 (14.8)	152 (13.5)	119 (16.3)	45 (18.3)	7 (16.3)
Emergency surgery	900 (12.0)	49 (15.4)	96 (12.7)	246 (14.3)	166 (11.5)	131 (11.9)	137 (12.2)	60 (8.2)	14 (5.7)	1 (2.3)
Non-traumatic cerebral pathology	477 (6.4)	38 (11.9)	67 (8.9)	104 (6.0)	84 (5.8)	52 (4.7)	68 (6.0)	48 (6.6)	12 (4.9)	4 (9.3)
Multiple trauma without head injury	141 (1.9)	12 (3.8)	28 (3.7)	39 (2.3)	25 (1.7)	14 (1.3)	18 (1.6)	5 (0.7)	0 (0.0)	0 (0.0)
Multiple trauma with head injury	128 (1.7)	15 (4.7)	24 (3.2)	36 (2.1)	18 (1.2)	18 (1.6)	9 (0.8)	7 (1.0)	1 (0.4)	0 (0.0)
Isolated head injury	180 (2.4)	10 (3.1)	29 (3.8)	43 (2.5)	30 (2.1)	20 (1.8)	29 (2.6)	13 (1.8)	4 (1.6)	2 (4.7)
Other	929 (12.4)	53 (16.6)	109 (14.4)	250 (14.5)	186 (12.9)	128 (11.7)	107 (9.5)	71 (9.7)	24 (9.8)	1 (2.3)
NIV (*n*, %)	1854 (24.8)	71 (22.3)	155 (20.5)	383 (22.2)	375 (25.9)	298 (27.2)	306 (27.2)	200 (27.4)	53 (21.5)	13 (30.2)
Intubation (*n*, %)	3804 (50.8)	165 (51.7)	421 (55.7)	885 (51.4)	731 (50.6)	510 (46.5)	551 (48.9)	357 (48.9)	159 (64.6)	25 (58.1)
Vasoactive drugs (*n*, %)	4400 (58.8)	173 (54.2)	436 (57.7)	1004 (58.3)	837 (57.9)	646 (58.9)	677 (60.1)	436 (59.7)	162 (65.9)	29 (67.4)
Renal replacement therapy (*n*, %)	820 (11.0)	24 (7.5)	72 (9.5)	208 (12.1)	165 (11.4)	101 (9.2)	126 (11.2)	76 (10.4)	43 (17.5)	5 (11.6)
Life sustaining treatment limitation										
None	4930 (65.8)	239 (74.9)	571 (75.5)	1236 (71.7)	982 (67.9)	688 (62.7)	669 (59.4)	370 (50.7)	154 (62.6)	21 (48.8)
Withdrawal	1108 (14.8)	39 (12.2)	107 (14.2)	234 (13.6)	194 (13.4)	166 (15.1)	183 (16.2)	135 (18.5)	39 (15.9)	11 (25.6)
Withholding	1449 (19.4)	41 (12.9)	78 (10.3)	253 (14.7)	270 (18.7)	243 (22.2)	275 (24.4)	225 (30.8)	53 (21.5)	11 (25.6)
Length of stay in the ICU, days, non-survivors (median [IQR])	3.00 [1.00, 7.93]	5.10 [1.87, 0.82]	3.00 [0.67, 8.85]	3.30 [1.00, 8.00]	3.80 [1.10, 9.00]	2.50 [1.00, 7.20]	3.00 [1.00, 7.00]	2.20 [0.80, 6.05]	3.00 [1.00, 8.20]	2.30 [0.80, 5.47]
Length of stay in the ICU, days, survivors (median [IQR])	3.30 [1.70, 7.00]	3.80 [1.80, 8.00]	3.50 [1.80, 6.93]	3.60 [1.80, 7.82]	3.50 [1.70, 7.00]	3.15 [1.50, 6.53]	3.00 [1.60, 6.23]	3.00 [1.35, 6.25]	6.20 [2.00,15.00]	3.70 [1.00,12.50]
Death in the ICU (*n*, %)	2024 (27.0)	60 (18.8)	188 (24.9)	403 (23.4)	369 (25.5)	273 (24.9)	331 (29.4)	259 (35.5)	113 (45.9)	28 (65.1)
Death in 30-day follow-up (*n*, %)	2985 (39.9)	91 (28.5)	254 (33.6)	576 (33.4)	545 (37.7)	416 (37.9)	513 (45.5)	406 (55.6)	149 (60.6)	35 (81.4)

*IQR* interquartile range, *ICU* intensive care unit, *NIV* non-invasive ventilation, *SOFA* Sequential Organ Failure Assessment

**Fig. 1:**
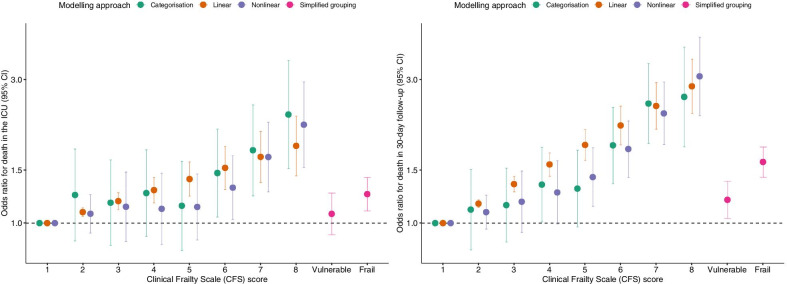
The impact of modelling approach on the association between frailty and short-term mortality. Simplified grouping used CFS < 4 as a reference, with vulnerability defined as a CFS = 4 and frailty as a CFS ≥ 5; CFS = 1 was used as a reference in the three other modelling approaches shown in the figure. Odds ratios obtained after dichotomisation of the CFS score are described in the text. The relationship between the CFS score and mortality was nonlinear in both models (*p* values < 0.01). Adjusted for age, sex, reason for admission to the ICU, and admission SOFA score

Results from both univariate (Additional file 1: Figure S4) and multivariable logistic regression models provided evidence that the relationship between the CFS score and mortality within 30 days is not linear (Fig. 1, Additional file 1: Figure S5).

The OR for ICU mortality was statistically significantly different from unity at a CFS score of 6 using both the categorical and nonlinear modelling approach based on restricted cubic splines. In case of 30-day mortality, an apparent increase in the odds of death at a CFS score equal to 6 was also apparent, though the lower boundary of the 95% confidence interval (CI) surpassed unity already at a CFS score of 4 and 5 using the categorical and nonlinear approach respectively. Evidence that vulnerability (CFS 4 vs. CFS < 4) is associated with an increased mortality was sufficient only in 30-day follow-up.

The magnitude of the estimated effect of frailty on mortality was greater in the 30-day mortality logistic regression compared to the ICU mortality model, and the slope of increase in the odds of death was steeper with increasing CFS scores. When patients were dichotomised as either frail (CFS ≥ 5) or not frail (CFS < 5), frailty was associated with an OR for mortality in the ICU and within 30 days equal to 1.22 (95% CI 1.09–1.36) and 1.50 (95% CI 1.35–1.66), respectively.

The fraction of new information from the categorical CFS score over and above data on patient’s age, sex, reason for admission to the ICU, and the SOFA score across different modelling strategies is shown in Table 2.

**Table 2:** Fraction of new prognostic information from including frailty in the model

	Fraction of new information		*p* value (likelihood ratio test)	
	ICU mortality model (%)	30-day mortality model (%)	ICU mortality model	30-day mortality model
Categorisation	3	9	< 0.001	< 0.001
Linear	2	8	< 0.001	< 0.001
Nonlinear	3	9	< 0.001	< 0.001
Simplified grouping	1	6	0.002	< 0.001
Dichotomisation	1	5	< 0.001	< 0.001

**Fig. 2:**
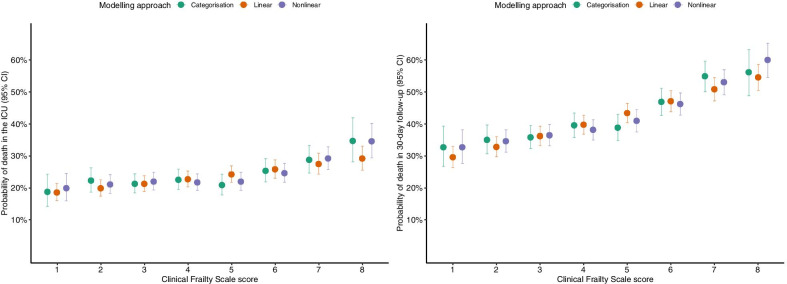
The impact of modelling approach on the model-calculated risk of death. Adjusted probabilities displayed for an 84-year-old male admitted due to respiratory reasons with a SOFA score equal to 7

Categorical and nonlinear modelling of the CFS was associated with the highest information yield, and dichotomising patients into either frail or not frail led to the greatest loss of information in both the ICU and 30-day mortality models. Likelihood ratio tests indicated that the addition of frailty significantly improves model fit in all cases (all *p* values < 0.01). Results of sensitivity analyses based on the 9-point version of the CFS score are presented in Additional file 1: Figures S6 and S7 and Table S3. The adjusted probability of death across different CFS scores is shown in Fig. 2.

## Discussion

We found that the relationship between frailty and 30-day mortality is not linear in patients ≥ 80 years old acutely admitted to the ICU. Using separate logistic regression models, we showed that the prognostic contribution of frailty was larger in case of 30-day mortality compared to ICU mortality. The association between the CFS score and mortality was highly dependent on how the score was handled in statistical analysis. Defining frailty as a CFS ≥ 5 led to a substantial underestimation of the odds of death for patients whose CFS score was equal to 7 or 8 and resulted in a loss of more than 50% of new prognostic information compared to using all levels of the 9-point Clinical Frailty Scale. The categorical and nonlinear approach to modelling frailty best captured the relationship between the CFS score and mortality and should be preferred over the other inspected methods of analysing frailty in future studies evaluating mortality in this population of patients.

A scoping review of the utilisation of the CFS score in the literature showed that 40 out of 58 papers (68.9%) using a threshold value of the CFS score to define frailty were based on a cut-off of 5, with 25.9% of publications based on a threshold of 4 and only 3.4% labelling patients with a CFS score ≥ 6 as frail [13]. Results of large registry-based study describing differences in outcomes of critically ill patients with pneumonia depending on their level of frailty conducted recently did not support a CFS ≥ 5 threshold to guide ICU admission [16]. The authors noted little difference between vulnerable, mild, and moderate frailty categories in terms of the adjusted risk of mortality. Another interesting finding was that the CFS score behaved differently in patients ≥ 65 years old than in younger adults. The observed increase in mortality with increasing CFS scores was apparently linear in the whole cohort and gradually accelerating in the older subgroup.

Results from a combined database of patients ≥ 80 years old enrolled in the VIP1 and VIP2 studies corroborate the results of Darvall and colleagues in the sense that there is little evidence of a difference in outcomes between patients whose CFS score ranges between 1 and 5 after adjustment for basic patient characteristics and that a threshold value of CFS ≥ 5 is not justified as a marker of frailty in the context of mortality within 30 days from admission to the ICU. Possibly thanks to the larger sample size, we were able to confirm that a CFS score of 6, not 7, is already associated with an increased risk of death. This minor discrepancy may either reflect a higher statistical power in current analyses or stem from the fact that our study enrolled a broad spectrum of patients admitted to the ICU and was not focused exclusively on patients with pneumonia. Unlike Darvall and colleagues, we found evidence that the relationship between frailty as described by the CFS score and mortality is nonlinear, which is in line with previous theoretical considerations about the expected character of increase in mortality with accumulating deficits [21].

The association between frailty and mortality was more pronounced in 30-day follow-up than when assessed up to the moment of death or discharge from the ICU. Several factors may underpin this finding, yet the most likely explanation is that despite surviving the acute phase of critical illness, frail patients ultimately reach a point of exhaustion of their functional reserves and deteriorate as a result of the initial stressor. It needs to be highlighted that despite not finding a clear association between lower CFS scores and mortality, it is reasonable to believe that patients with wild milder frailty fare worse in terms of functional outcomes and quality of life in long-term observation after being admitted to the ICU [22–24].

A more general claim that can be made based on the results of our study is that any attempts to define frailty as a condition that is either present or absent are destined to fail. Despite the conceptual ease and convenience of treating patients as fit or frail both from a researcher’s and practicing clinician’s perspective, we must not forget that frailty is a continuum. Loss of prognostic information is inevitable whenever arbitrary grouping of patients takes place, particularly when trying to transform a robust score such as the CFS into a binary screening tool. In practice, decisions such as whether to admit a patient to the ICU or not have to be made at some point based on a context-specific threshold [25]. Nevertheless, efforts should be made to present the clinician with the most accurate and comprehensive picture of the patient’s prospects and let her or him utilise the full potential of analysed data (i.e. the probability of an event based on a granular assessment of patient’s frailty, not merely the information whether an arbitrary CFS threshold had been crossed).

Even with the best available estimates of prognosis based on robust statistical models, the decision-making process in the context of intensive care provision for older adults will remain a multidimensional challenge shaped by a plethora of contextual and temporal factors that are difficult to capture in a set of static regression coefficients. The evidence to inform risk–benefit assessment on admission to the ICU and further into the course of critical illness in vulnerable populations is accumulating, with more detailed data on functional outcomes in long-term observation to come in the near future. It is in our patients’ interest that we transform this evidence into policies efficiently, understanding the consequences of choices made at the stage of data analysis. If the adjusted probability of death within 30 days from admission to the ICU with a CFS score of 5, 6, and 7 is estimated at 39%, 47%, and 55%, as illustrated in our study, where should we draw a demarcation line for prognostically important frailty, if at all? Before attempting to answer this question, one must first acknowledge the variability in outcomes between particular categories of the CFS score that arbitrary cut-offs unfortunately discard altogether.

In observational studies attempting to adjust for confounding by frailty or quantify its impact on outcomes, using the CFS score as either a categorical variable comprising all the original levels or a continuous variable while allowing for nonlinearities in the estimated effect of frailty on outcome is likely to lead to the least amount of residual confounding and improve inference compared to simpler approaches to modelling frailty such as dichotomisation or forced linearity [26, 27].

This study has several limitations. First, ICUs included in the VIP1 and VIP2 studies participated voluntarily and were not sampled at random. Second, informed consent from patients or their legal representatives had to be collected before enrolment in most participating countries due to national differences in data protection regulations which may affect the generalizability of our results to the entire population of acutely admitted patients. Third, a small fraction of patients had still been hospitalized in the ICU after 30 days from admission, thus increasing the uncertainty about actual mortality rates observed within our study. Fourth, the pragmatic nature of the VIP1 and VIP2 studies precluded routine collection of more detailed data on patients’ chronic health status prior to admission to the ICU, potentially leaving a substantial part of variation in the modelled outcome unexplained and possibly inflating the fraction of new prognostic information from frailty. Fifth, we focused exclusively on mortality and did not trace post-discharge health trajectories of VIPs (e.g. in terms of functional capacity, cognitive impairment, and mental health) in long-term observation.

The strength of our study is that we prospectively enrolled a large, contemporary cohort of consecutive patients ≥ 80 years old admitted to ICUs in 26 countries for various reasons. The collection of standardized data from nearly 8000 VIPs and the use of contemporary statistical modelling techniques provided an ample opportunity to answer the question about the optimal approach to analysing frailty in this population of patients.

## Conclusions

In conclusion, frailty contributes up to 9% of new prognostic information about 30-day mortality in the presence of basic patient characteristics. We showed that the increase in the risk of death within 30 days from admission to the ICU is mild at best in lower categories of the CFS score and more abrupt in patients whose CFS score is equal to 6 or greater. To accurately describe the relationship between frailty and short-term mortality, future studies should analyse the CFS score either as a nonlinear continuous variable or a categorical variable while retaining all the original levels of frailty.

## Supplementary Information

**Additional file 1.** Figures S1–S3 and Tables S1 and S2.

## Data Availability

Data collected for the study (anonymized database) as well as additional related documents (study protocol, statistical analysis plan, informed consent form) will be made available for the reviewers and editors of the Journal upon request. Relevant documents will be made available by study investigators via password-protected data-sharing web page.
