# Supplementary material for: Relationship between the Clinical Frailty Scale and short-term mortality in patients ≥ 80 years old acutely admitted to the ICU: a prospective cohort study
Source: Crit Care. 2021 Jul 1;25:231. doi: 10.1186/s13054-021-03632-3 (PMC8247215; doi:10.1186/s13054-021-03632-3)
Supplement: Supplementary file 1 — Additional file 1. Figures S1–S3 and Tables S1 and S2. [file 13054_2021_3632_MOESM1_ESM.docx]

**SUPPLEMENTARY MATERIAL**

**Relationship between the Clinical Frailty Scale and short-term mortality in patients ≥80 years old acutely admitted to the ICU – a prospective cohort study**

**TABLE OF CONTENTS:**

**Supplementary Figure 1.** Detailed description of missing variables: entire dataset. **(Page 2)**

**Supplementary Figure 2.** Detailed description of missing variables: stratified by country. **(Page 3)**

**Supplementary Figure 3.** The number of patients still hospitalised in the ICU within 30 days

from admission stratified by country. **(Page 4)**

**Supplementary Table 1.** Characteristics of complete and incomplete observations in terms

of survival status within 30 days from admission to the ICU. **(Page 5)**

**Supplementary Table 2.** Characteristics of patients who were discharged from the ICU

and patients still hospitalised in the ICU at day 30. **(Page 6)**

**Supplementary Figure 4**. The impact of modelling approach on the association between frailty

and short-term mortality – univariate logistic regression, 8-point variant of the Clinical Frailty Scale. **(Page 7)**

**Supplementary Figure 5**. Relationship between frailty and short-term mortality after admission to the ICU on log odds scale, 8-point variant of the Clinical Frailty Scale. (**Page 7**)

**Supplementary Figure 6**. The impact of modelling approach on the association between frailty and short-term mortality – univariate logistic regression, 9-point variant of the Clinical Frailty Scale. **(Page 8)**

**Supplementary Figure 7**. The impact of modelling approach on the association between frailty and short-term mortality – multivariable logistic regression, 9-point variant of the Clinical Frailty Scale. **(Page 8)**

**Supplementary Table 3.** Fraction of new prognostic information from including frailty in the model – 9-point variant of the Clinical Frailty Scale. **(Page 9)**

**Supplementary Table 4.** STROBE checklist **(Page 10)**

**Supplementary Table 5.** VIP1 & VIP2 collaborators list **(Page 12)**

**Supplementary Figure 1.** Detailed description of missing variables: entire dataset.

**Footnote:** Blue – complete observations, red – missing data.

**Supplementary Figure 2.** Detailed description of missing variables: stratified by country.

**Supplementary Figure 3.** The number of patients still hospitalised in the ICU within 30 days from admission stratified by country.

**Supplementary Table 1.** Characteristics of complete and incomplete observations in terms of survival status within 30 days from admission to the ICU.

|  | Complete observations | Lost to follow-up |
| --- | --- | --- |
| Number of patients | 7723 | 450 |
| Reason for admission (n, %) |  |  |
| Respiratory failure | 1841 (24.1) | 97 (23.5) |
| Circulatory failure | 1074 (14.1) | 47 (11.4) |
| Combined respiratory/circulatory failure | 893 (11.7) | 57 (13.8) |
| Sepsis | 1019 (13.3) | 42 (10.2) |
| Emergency surgery | 915 (12.0) | 37 (9.0) |
| Non-traumatic cerebral pathology | 487 (6.4) | 32 (7.7) |
| Multiple trauma without head injury | 143 (1.9) | 11 (2.7) |
| Multiple trauma with head injury | 134 (1.8) | 13 (3.1) |
| Isolated head injury | 183 (2.4) | 14 (3.4) |
| Other | 952 (12.5) | 63 (15.3) |
| Male sex (n, %) | 4060 (52.6) | 228 (50.7) |
| Age, years (median [IQR]) | 84.00 [81.00, 87.00] | 83.00 [81.00, 86.00] |
| SOFA score (median [IQR]) | 7.00 [4.00, 10.00] | 6.00 [4.00, 9.00] |
| CFS (median [IQR]) | 4.00 [3.00, 6.00] | 4.00 [3.00, 6.00] |
| NIV (n, %) | 1900 (24.6) | 109 (24.6) |
| Intubation (n, %) | 3927 (50.9) | 237 (53.0) |
| Vasoactive drugs (n, %) | 4495 (58.2) | 202 (45.4) |
| Renal replacement therapy (n, %) | 840 (10.9) | 33 (7.5) |
| Length of stay in the ICU, hours (median [IQR]) | 76.00 [35.00, 174.00] | 72.50 [30.00, 160.50] |

**Supplementary Table 2.** Characteristics of patients who were discharged from the ICU and patients still hospitalised in the ICU at day 30.

|  | LOS ≤30 days | LOS >30 days |
| --- | --- | --- |
| Number of patients | 7263 | 224 |
| Reason for admission (n, %) |  |  |
| Respiratory failure | 1744 (24.0) | 65 (29.0) |
| Circulatory failure | 1046 (14.4) | 11 (4.9) |
| Combined respiratory/circulatory failure | 834 (11.5) | 40 (17.9) |
| Sepsis | 958 (13.2) | 34 (15.2) |
| Emergency surgery | 882 (12.1) | 18 (8.0) |
| Non-traumatic cerebral pathology | 459 (6.3) | 18 (8.0) |
| Multiple trauma without head injury | 136 (1.9) | 5 (2.2) |
| Multiple trauma with head injury | 117 (1.6) | 11 (4.9) |
| Isolated head injury | 175 (2.4) | 5 (2.2) |
| Other | 912 (12.6) | 17 (7.6) |
| Male sex (n, %) | 3802 (52.3) | 132 (58.9) |
| Age, years (median [IQR]) | 84.00 [81.00, 87.00] | 83.00 [81.00, 85.00] |
| SOFA score (median [IQR]) | 7.00 [4.00, 10.00] | 9.00 [6.00, 11.00] |
| CFS (median [IQR]) | 4.00 [3.00, 6.00] | 4.00 [3.00, 6.00] |
| NIV (n, %) | 1798 (24.8) | 56 (25.0) |
| Intubation (n, %) | 3595 (49.5) | 209 (93.3) |
| Vasoactive drugs (n, %) | 4198 (57.8) | 202 (90.2) |
| Renal replacement therapy (n, %) | 753 (10.4) | 67 (29.9) |

**Supplementary Figure 4**. The impact of modelling approach on the association between frailty and short-term mortality – univariate logistic regression, 8-point variant of the Clinical Frailty Scale.

**Supplementary Figure 5**. Relationship between frailty and short-term mortality after admission to the ICU on log odds scale, 8-point variant of the Clinical Frailty Scale.

**Supplementary Figure 6**. The impact of modelling approach on the association between frailty and short-term mortality – univariate logistic regression, 9-point variant of the Clinical Frailty Scale.

**Supplementary Figure 7**. The impact of modelling approach on the association between frailty and short-term mortality – multivariable logistic regression, 9-point variant of the Clinical Frailty Scale.

**Supplementary Table 3.** Fraction of new prognostic information from including frailty in the model – 9-point variant of the Clinical Frailty Scale.

|  | **Fraction of new information** | | **p-value (likelihood ratio test)** | |
| --- | --- | --- | --- | --- |
|  | ICU mortality model | 30-day mortality model | ICU mortality model | 30-day mortality model |
| Categorisation | 4% | 11% | <0.001 | <0.001 |
| Linear | 3% | 9% | <0.001 | <0.001 |
| Nonlinear | 4% | 10% | <0.001 | <0.001 |
| Simplified grouping | 1% | 6% | <0.001 | <0.001 |
| Dichotomisation | 1% | 5% | <0.001 | <0.001 |

**Supplementary Table 4.** STROBE checklist

|  | Item No | Recommendation | Page No |
| --- | --- | --- | --- |
| **Title and abstract** | 1 | (*a*) Indicate the study’s design with a commonly used term in the title or the abstract |  |
|  |  | (*b*) Provide in the abstract an informative and balanced summary of what was done and what was found | 1-3 |
| Introduction | | | |
| Background/rationale | 2 | Explain the scientific background and rationale for the investigation being reported | 4-5 |
| Objectives | 3 | State specific objectives, including any prespecified hypotheses | 5 |
| Methods | | | |
| Study design | 4 | Present key elements of study design early in the paper | 5 |
| Setting | 5 | Describe the setting, locations, and relevant dates, including periods of recruitment, exposure, follow-up, and data collection | 5 |
| Participants | 6 | (*a*) Give the eligibility criteria, and the sources and methods of selection of participants. Describe methods of follow-up |  |
|  |  | (*b*) For matched studies, give matching criteria and number of exposed and unexposed | 5 |
| Variables | 7 | Clearly define all outcomes, exposures, predictors, potential confounders, and effect modifiers. Give diagnostic criteria, if applicable | 6-8 |
| Data sources/ measurement | 8* | For each variable of interest, give sources of data and details of methods of assessment (measurement). Describe comparability of assessment methods if there is more than one group | 6-8 |
| Bias | 9 | Describe any efforts to address potential sources of bias | 6-8 |
| Study size | 10 | Explain how the study size was arrived at | 5 |
| Quantitative variables | 11 | Explain how quantitative variables were handled in the analyses. If applicable, describe which groupings were chosen and why | 6-8 |
| Statistical methods | 12 | (*a*) Describe all statistical methods, including those used to control for confounding |  |
|  |  | (*b*) Describe any methods used to examine subgroups and interactions | 6-8 |
|  |  | (*c*) Explain how missing data were addressed |  |
|  |  | (*d*) If applicable, explain how loss to follow-up was addressed |  |
|  |  | (*e*) Describe any sensitivity analyses |  |
| Results | | |  |
| Participants | 13* | (a) Report numbers of individuals at each stage of study—eg numbers potentially eligible, examined for eligibility, confirmed eligible, included in the study, completing follow-up, and analysed | 8 |
|  |  | (b) Give reasons for non-participation at each stage |  |
|  |  | (c) Consider use of a flow diagram |  |
| Descriptive data | 14* | (a) Give characteristics of study participants (eg demographic, clinical, social) and information on exposures and potential confounders | 8-9, T1, SM |
|  |  | (b) Indicate number of participants with missing data for each variable of interest |  |
|  |  | (c) Summarise follow-up time (eg, average and total amount) |  |
| Outcome data | 15* | Report numbers of outcome events or summary measures over time | 8-9, T1, SM |
| Main results | 16 | (*a*) Give unadjusted estimates and, if applicable, confounder-adjusted estimates and their precision (eg, 95% confidence interval). Make clear which confounders were adjusted for and why they were included | 8-9, T2, F1-2, SM |
|  |  | (*b*) Report category boundaries when continuous variables were categorized | 7 |
|  |  | (*c*) If relevant, consider translating estimates of relative risk into absolute risk for a meaningful time period |  |
| Other analyses | 17 | Report other analyses done—eg analyses of subgroups and interactions, and sensitivity analyses | 8-9, SM |
| Discussion |  |  |  |
| Key results | 18 | Summarise key results with reference to study objectives | 10 |
| Limitations | 19 | Discuss limitations of the study, taking into account sources of potential bias or imprecision. Discuss both direction and magnitude of any potential bias | 12-13 |
| Interpretation | 20 | Give a cautious overall interpretation of results considering objectives, limitations, multiplicity of analyses, results from similar studies, and other relevant evidence | 13 |
| Generalisability | 21 | Discuss the generalisability (external validity) of the study results | 11-13 |
| Other information |  |  |  |
| Funding | 22 | Give the source of funding and the role of the funders for the present study and, if applicable, for the original study on which the present article is based | 14 |

**Supplementary Table 5.** VIP1 & VIP2 collaborators list

| **First initials and surnames of VIP1 collaborators** |
| --- |
| R Schmutz |
| F Wimmer |
| P Eller |
| M Joannidis |
| P De Buysscher |
| N De Neve |
| S Oeyen |
| W Swinnen |
| B Bollen Pinto |
| P Abraham |
| L Hergafi |
| JC Schefold |
| E Biskup |
| P Piza |
| I Taliadoros |
| J Fjølner |
| N Dey |
| C Sølling |
| BS Rasmussen |
| S Christensen |
| X Forceville |
| G Besch |
| H Mentec |
| P Michel |
| P Mateu |
| P Michel |
| L Vettoretti |
| J Bourenne |
| N Marin |
| M Guillot |
| N Aissaoui |
| C Goulenok |
| N Thieulot-Rolin |
| J Messika |
| L Lamhaut |
| B Guidet |
| C Charron |
| A Lauten |
| AL Sacher |
| T Brenner |
| M Franz |
| F Bloos |
| H Ebelt |
| S J Schaller |
| K Fuest |
| C Rabe |
| T Dieck |
| S Steiner |
| T Graf |
| AM Nia |
| C Jung |
| RA Janosi |
| P Meybohm |
| P Simon |
| S Utzolino |
| T Rahmel |
| E Barth |
| C Jung |
| M Schuster |
| Z Aidoni |
| S Aloizos |
| P Tasioudis |
| K Lampiri |
| V Zisopoulou |
| I Ravani |
| E Pagaki |
| A Antoniou |
| TA Katsoulas |
| A Kounougeri |
| G Marinakis |
| F Tsimpoukas |
| A Spyropoulou |
| P Zygoulis |
| A Kyparissi |
| M Gupta |
| M Gurjar |
| IM Maji |
| I Hayes |
| B Marsh |
| Y Kelly |
| A Westbrook |
| G Fitzpatrick |
| D Maheshwari |
| C Motherway |
| G Negri |
| S Spadaro |
| G Nattino |
| M Pedeferri |
| A Boscolo |
| S Rossi |
| G Calicchio |
| L Cubattoli |
| G Di Lascio |
| M Barbagallo |
| F Berruto |
| D Codazzi |
| A Bottazzi |
| P Fumagalli |
| G Negro |
| G Lupi |
| F Savelli |
| GA. Vulcano |
| R Fumagalli |
| A Marudi |
| U Lefons |
| R Lembo |
| M Babini |
| A Paggioro |
| V Parrini |
| M Zaccaria |
| S Clementi |
| C Gigliuto |
| F Facondini |
| S Pastorini |
| S Munaron |
| I Calamai |
| A Bocchi |
| A Adorni |
| MG Bocci |
| A Cortegiani |
| T Casalicchio |
| S Mellea |
| E Graziani |
| M Barattini |
| E Brizio |
| M Rossi |
| M Hahn |
| H Flaatten |
| N Kemmerer |
| HF Strietzel |
| K Dybwik |
| T Legernaes |
| P Klepstad |
| EB Olaussen |
| KI Olsen |
| OM Brresen |
| G Bjorsvik |
| FH Andersen |
| S Maini |
| L Fehrle |
| M Czuczwar |
| P Krawczyk |
| M Ziętkiewicz |
| ŁR Nowak |
| K Kotfis |
| K Cwyl |
| R Gajdosz |
| J Biernawska |
| R Bohatyrewicz |
| R Gawda |
| P Grudzień |
| P Nasiłowski |
| N Popek |
| W Cyrankiewicz |
| K Wawrzyniak |
| M Wnuk |
| D Maciejewski |
| D Studzińska |
| M Żukowski |
| S Bernas |
| M Piechota |
| W Szczeklik |
| I Nowak-Kózka |
| J Fronczek |
| M Serwa |
| W Machała |
| J Stefaniak |
| M Wujtewicz |
| P Maciejewski |
| M Szymkowiak |
| B Adamik |
| K Polok |
| J Górka |
| N Catorze |
| MC Branco |
| N Barros |
| I Barros |
| A Krystopchuk |
| T Honrado |
| C Sousa |
| F Munoz |
| M Rebelo |
| R Gomes |
| J Nunes |
| C Dias |
| AM Fernandes |
| C Petrisor |
| B Constantin |
| V Belskiy |
| B Boskholov |
| E Rodriguez |
| G Aguilar |
| G Masdeu |
| MI Jaimes |
| AP Mira |
| MA Bodi |
| JAB Mendoza |
| S López-Cuenca |
| MH Guzman |
| J Rico-Feijoo |
| M Ibarz |
| J Trenado Alvarez |
| R Kawati |
| J Sivik |
| J Nauska |
| D Smole |
| F Parenmark |
| J Lyrén |
| K Rockstroh |
| S Rydén |
| M Spångfors |
| M Strinnholm |
| S Walther |
| L De Geer |
| P Nordlund |
| S Pålsson |
| H Zetterquist |
| A Nilsson |
| K Thiringer |
| M Jungner |
| B Bark |
| B Nordling |
| H Sköld |
| C Brorsson |
| S Persson |
| A Bergström |
| J Berkius |
| J Holmström |
| I van Dijk |
| LEM. van Lelyveld-Haas |
| T Jansen |
| F Nooteboom |
| PHJ van der Voort |
| D de Lange |
| W Dieperink |
| MC de Waard |
| AGE de Smet |
| L Bormans |
| T Dormans |
| G Dempsey |
| SJ Mathew |
| AS Raj |
| I Grecu |
| J Cupitt |
| T Lawton |
| R Clark |
| M Popescu |
| N Spittle |
| M Faulkner |
| A Cowton |
| P Williams |
| E Elloway |
| M Reay |
| S Chukkambotla |
| R Kumar |
| N Al-Subaie |
| L Kent |
| T Tamm |
| I Kajtor |
| K Burns |
| R Pugh |
| M Ostermann |
| E Kam |
| H Bowyer |
| N Smith |
| M Templeton |
| J Henning |
| K Goffin |
| R Kapoor |
| S Laha |
| P Chilton |
| W Khaliq |
| A Crayford |
| S Coetzee |
| M Tait |
| W Stoker |
| M Gimenez |
| A Pope |
| J Camsooksai |
| D Pogson |
| K Quigley |
| J Ritzema |
| A Hormis |
| C Boulanger |
| M Balasubramaniam |
| L Vamplew |
| K Burt |
| D Martin |
| I Grecu |
| J Craig |
| J Prowle |
| N Doyle |
| J Shelton |
| C Scott |
| P Donnison |
| S Shelton |
| C Frey |
| C Ryan |
| D Spray |
| C Ryan |
| V Barnes |
| K Barnes |
| S Ridgway |
| R Saha |
| L Kent |
| T Clark |
| J Wood |
| C Bolger |
| C Bassford |
| A Cowton |
| J Lewandowski |
| X Zhao |
| S Humphreys |
| S Dowling |
| N Richardson |
| A Burtenshaw |
| C Stevenson |
| D Wilcock |
| Y Nalapko |
| **First initials and surnames of VIP2 collaborators** |
| M Joannidis |
| P Eller |
| R Helbok |
| R Schmutz |
| J Nollet |
| N de Neve |
| P De Buysscher |
| S Oeyen |
| W Swinnen |
| M Mikačić |
| A Bastiansen |
| A Husted |
| BES Dahle |
| C Cramer |
| C Sølling |
| D Ørsnes |
| J Edelberg Thomsen |
| JJ Pedersen |
| M Hummelmose Enevoldsen |
| T Elkmann |
| A Kubisz-Pudelko |
| A Pope |
| A Collins |
| AS Raj |
| C Boulanger |
| C Frey |
| C Hart |
| C Bolger |
| D Spray |
| G Randell |
| H Filipe |
| ID Welters |
| I Grecu |
| J Evans |
| J Cupitt |
| J Lord |
| J Henning |
| J Jones |
| J Ball |
| J North |
| K Salaunkey |
| L Ortiz-Ruiz De Gordoa |
| L Bell |
| M Balasubramaniam |
| M Vizcaychipi |
| M Faulkner |
| M Mupudzi |
| M Lea-Hagerty |
| M Reay |
| M Spivey |
| N Love |
| N Spittle |
| N White |
| P Williams |
| P Morgan |
| P Wakefield |
| R Savine |
| R Jacob |
| R Innes |
| R Kapoor |
| S Humphreys |
| S Rose |
| S Dowling |
| S Leaver |
| T Mane |
| T Lawton |
| V Ogbeide |
| W Khaliq |
| Y Baird |
| A Romen |
| A Galbois |
| B Guidet |
| C Vinsonneau |
| C Charron |
| D Thevenin |
| E Guerot |
| G Besch |
| G Savary |
| H Mentec |
| JL Chagnon |
| JP Rigaud |
| JP Quenot |
| J Castaneray |
| J Rosman |
| J Maizel |
| K Tiercelet |
| L Vettoretti |
| MM Hovaere |
| M Messika |
| M Djibré |
| N Rolin |
| P Burtin |
| P Garcon |
| S Nseir |
| X Valette |
| C Rabe |
| E Barth |
| H Ebelt |
| K Fuest |
| M Franz |
| M Horacek |
| M Schuster |
| P Meybohm |
| R Romano Bruno |
| S Allgäuer |
| S Dubler |
| SJ Schaller |
| S Schering |
| S Steiner |
| T Dieck |
| T Rahmel |
| T Graf |
| A Koutsikou |
| A Vakalos |
| B Raitsiou |
| EN Flioni |
| E Neou |
| F Tsimpoukas |
| G Papathanakos |
| G Marinakis |
| I Koutsodimitropoulos |
| K Aikaterini |
| N Rovina |
| S Kourelea |
| T Polychronis |
| V Zidianakis |
| V Konstantinia |
| Z Aidoni |
| B Marsh |
| C Motherway |
| C Read |
| I Martin-Loeches |
| A Neville Cracchiolo |
| A Morigi |
| I Calamai |
| S Brusa |
| A Elhadi |
| A Tarek |
| A Khaled |
| H Ahmed |
| W Ali Belkhair |
| AD Cornet |
| D Gommers |
| D de Lange |
| E van Boven |
| J Haringman |
| L Haas |
| L van den Berg |
| O Hoiting |
| P de Jager |
| R T. Gerritsen |
| T Dormans |
| W Dieperink |
| A Breidablik |
| A Slapgard |
| AK Rime |
| B Jannestad |
| B Sjøbøe |
| E Rice |
| FH Andersen |
| HF Strietzel |
| JP Jensen |
| J Langørgen |
| K Tøien |
| K Strand |
| M Hahn |
| P Klepstad |
| A Biernacka |
| A Kluzik |
| B Kudlinski |
| D Maciejewski |
| D Studzińska |
| H Hymczak |
| J Stefaniak |
| J Solek-Pastuszka |
| J Zorska |
| K Cwyl |
| ŁJ Krzych |
| M Zukowski |
| M Lipińska-Gediga |
| M Pietruszko |
| M Piechota |
| M Serwa |
| M Czuczwar |
| M Ziętkiewicz |
| N Kozera |
| P Nasiłowski |
| P Sendur |
| P Zatorski |
| P Galkin |
| R Gawda |
| U Kościuczuk |
| W Cyrankiewicz |
| W Gola |
| AF Pinto |
| AM Fernandes |
| AR Santos |
| C Sousa |
| I Barros |
| IA Ferreira |
| JB Blanco |
| JT Carvalho |
| J Maia |
| N Candeias |
| N Catorze |
| V Belskiy |
| A Lores |
| AP Mira |
| C Cilloniz |
| D Perez-Torres |
| E Maseda |
| E Rodriguez |
| E Prol-Silva |
| G Eixarch |
| G Gomà |
| G Aguilar |
| G Navarro Velasco |
| M Irazábal Jaimes |
| M Ibarz Villamayor |
| N Llamas Fernández |
| P Jimeno Cubero |
| S López-Cuenca |
| T Tomasa |
| A Sjöqvist |
| C Brorsson |
| F Schiöler |
| H Westberg |
| J Nauska |
| J Sivik |
| J Berkius |
| K Kleiven Thiringer |
| L De Geer |
| S Walther |
| F Boroli |
| JC Schefold |
| L Hergafi |
| P Eckert |
| I Yıldız |
| I Yovenko |
| Y Nalapko |
| R Pugh |
